# Treatment Outcomes and Clinicopathologic Characteristics of Triple-Negative Breast Cancer: A Report from Cancer Institute of Iran

**Published:** 2017-01-01

**Authors:** Mehrzad Mirzania, Seyed Reza Safaee, Farhad Shahi, Issa Jahanzad, Ghazal Zahedi, Reza Mehdizadeh

**Affiliations:** 1 Department of Medical Oncology, Cancer Research Center, Cancer Institute, Imam Khomeini Hospital Complex, Tehran University of Medical Sciences, Tehran, Iran; 2 Department of Pathology, Cancer Institute, Imam Khomeini Hospital Complex, Tehran University of Medical Sciences, Tehran, Iran; 3 Department of Internal Medicine, Imam Khomeini Hospital, Tehran University of Medical Sciences, Tehran, Iran; 4 Breast Cancer Research Center, Academic Center for Education Culture and Research (ACECR), Tehran, Iran

**Keywords:** Basal-like tumors, Triple-negative breast cancer, Iran

## Abstract

**Background:** Triple-negative breast cancers (TNBC) have a more aggressive course and are associated with poorer prognosis in comparison with other subtypes of breast cancer. One of the most common subtypes of TNBC is basal-like. The aim of this study was to investigate clinicopathological characteristics and clinical course of TNBC in Iranian women and compare them with other studies.

**Subjects and Methods:** Between March 2009 and February 2011, patients with breast cancer in Cancer Institute of Iran were selected and then followed-up for 2 years. Paraffin-embedded tumor block of all TNBC patients were evaluated for CK5/6 and EGFR using IHC method.

**Results:** Among 267 breast cancer patients, 60 cases with TNBC were identified (22.5%), 31 patients (51.7%) had basal-like and 29 patients (48.3%) had non-basal-like tumors. The median age of participants with TNBC was 49.6 years. Among our patients, 70% had positive lymph nodes.93.4% of all patients at the time of diagnosis were stage II or III and tumor size was at least 3 centimeters. No grade 1 TNBC was found in this study. During the follow-up period, there were 26 recurrences and 7 deaths.

**Conclusion:** The percentage of basal-like subtype among Iranian women with TNBC was lower compared to other studies, while bone metastases, clinical stage, lymph node involvement and tumor size were higher. Clinicopathological findings in basal and non-basal-like subgroups were not different, but the probability of lymph node involvement was more common in patients who were EGFR positive.

## Introduction

 In definition, TNBCs are negative or low in expression (≤1 percent) of the ER, PR and negative or low in expression of HER2 [0-1+ by IHC, or 2+ by IHC and in situ hybridization (ISH) negative].^1^ TNBCs account for approximately 20% of diagnosed cases of breast cancer.^2^ They present aggressively with rapid growth and are diagnosed clinically rather than radio logically.^3^ Triple-negative breast cancers are usually high grade in pathologic features, aggressive in clinical course and poorer prognosis compared with other breast cancer subtypes^3^^-^^6^ and are more common in women less than 40 years compared with hormone receptor- positive breast cancer.^7^^,^^8^ TNBCs and basal-like tumors are not synonymous. In one study, 71% of 172 triple-negative breast cancers were assigned to basal subtype.^9^ On the other hand, in an analysis of 160 tumors, 77% of basal-like tumors were triple-negative by IHC. TNBC tumors seem to be associated with poor outcomes, comparable to those of basal-like tumors that are not triple negative.^10^^,^^11^

Basal-like tumors are characterized by the genomic expression of the "basal cluster," a cluster of genes, including the epidermal growth factor receptor (EGFR also called HER1), basal cytokeratins 5/6 and c-Kit.^8^^,^^12^^-^^14^ Compared with other breast cancer subtypes, cells in the basal-like tumors have a higher degree of genetic instability and also a greater rate of loss of heterozygosity.^15^

Other subtypes of triple-negative breast cancer are immunomodulatory, mesenchymal stem cells, mesenchymal stem-like and luminal androgen subtypes.^16^

Despite the abundance of studies investigating molecular, clinical, epidemiological, therapeutic, and prognostic aspects of basal-like tumors, clinicopathological characteristics of basal-like and non-basal-like subtype of TNBC have never been described in the Iranian population. Iranian women with breast cancer tend to be diagnosed with the disease about one decade earlier than the women living in the Western countries^17^ and also suffering from poorer prognosis.^18^ Moreover, the clinicopathologyand immunohistochemistry of basal-like subtype in comparison with non-basal-like TNBC tumors have rarely been explored. The present study was thus designed and conducted to describe the prevalence, clinical and pathological characteristics of basal-like and non-basal-like TNBC patients in a large referral hospital in Tehran (Cancer Institute, Tehran University of medical Sciences, Tehran, Iran).

## SUBJECTS AND METHODS

 Between March 2009 and February 2011, medical records of all patients with the primary diagnosis of malignant breast tumor were retrieved. All patients within the study had undergone surgery and their tissue samples were available. Patients’ clinical information was extracted from medical records. Pathology reports were also retrieved and data regarding tumor size, grade, presence of lymph node involvement, vascular invasion, neural invasion, hormone receptor status (ER, PR) and HER2 were obtained. Patients were followed-up for a median of 24 months (ranging from 9 to 30 months).

In the two-year period, 267 patients with complete clinical, pathological, and receptor status reports were found, of whom 60 were diagnosed with TNBC. Formalin-fixed, paraffin-embedded blocks for this TNBC subset was retrieved and sent for further IHC analysis. All patients were seen at the clinic and last clinical status was recorded. Informed consent was obtained from all participants. Local hospital ethic committee approved the study protocol and supervised the data acquisition process. (Ethical code: 18210-51-03-91).

In the present study, two immunohistochemistry (IHC) markers, namely cytokeratin 5/6 (CK 5/6) and epidermal growth factor receptor (EGFR, HER 1) which are over expressed in basal-like tumors, were used to categorize breast tumors into basal-like and non-basal-like subtypes. We used monoclonal antibody kits manufactured by Dako (Dako North America Inc., Carpinteria, California). In this subset, as described by Carey et al. basal-like subtype was defined if tumors were positive for either CK 5/6, or EGFR. Tumors negative for both markers were designated non basal-like.^19^

Nielsen et al. compared IHC method against microarray and demonstrated that a panel of four markers (antibodies against ER, HER2, CK5/6, and EGFR) can be applied to identify basal-like tumors with a sensitivity and specificity of 76% and 100%, respectively.^20^

Processed samples were evaluated under light microscope by a single experienced pathologist blinded to the clinical data of the patient. EGFR positive staining was defined as any complete or partial staining of the cell membrane^21^ CK 5/6 positivity was defined as any cytoplasmic and/or membranous staining of the cells.^22^

## Results

 Two hundred and sixty-seven cases with complete ER, PR, and HER2 status were identified, of whom 60 (22.5%) were diagnosed with TNBC. The clinical and pathological characteristics of patients with TNBC are summarized in Table 1. The median age at the time of breast cancer diagnosis was 49.6 years. The pathologic subtypes were invasive-ductal carcinoma (95%), medullary carcinoma (3.3%) and invasive lobular carcinoma (1.7%). No grade 1 tumors were found. Neural and vascular invasion were reported in 53.3% and 63.3% of the evaluated specimens, respectively. The number of excised lymph nodes ranged from 1 to 25 and median lymph node positivity was 70%. During the follow-up period, recurrence was identified in 26 (43.2%); patients, of whom 22 (36.67%) had distant recurrences. Bones were the most frequent sites of metastasis, followed by lungs and brain. Patients were followed-up for a median of 24 months (ranging from 9 to 30 months) and 7 (11.67%) patients were died of breast cancer during this time period.

Lymph node positivity, clinical staging, bone metastases and tumor size were higher in our study compared with other studies (Table 1).

The subtypes of basal-like and non-basal-like were determined by IHC markers of CK 5/6 and EGFR and the results are displayed in Table 2.

Age at diagnosis, disease stage, lymph node involvement, size, type, grade of the tumor, and also the presence of neural or vascular invasion were not significantly different between basal and non-basal-like subtypes (Table 3). EGFR+ tumors were more likely to be lymph node positive (91.7% vs. 64.5%, p=0.045).

## Discussion

 Clinicopathological characteristics and outcomes were compared with other studies (Table 1).

In contrast to some reports from Iran,^17^ the mean age of the Iranian patients was similar to other studies.^3^^,^^6^^,^^31^^-^^32^ Clinical stage 1 in our patients was significantly less than other studies, while clinical stage 3 was more in this study (Table 1). Lymph node involvement at diagnosis time among Iranian women participated in this study was 70%, while it was lower in other studies and ranged from 38% to 62.7% (Table 1). Lymphovascular invasion in our study was 63%, while it was lower in other studies (26%-39.6%).^3^^,^^6^ The recurrence rate in our patients was similar to other studies^3^ but the location was different; bone recurrence was more common (Table 1).

The majority of studies that have pointed out to an unfavorable outcome for basal-like tumors have compared this subtype against ER positive subtypes of luminal A and B except TNBC tumors.^23^^-^^26^ Yamamoto et al. further explored the effect of basal-like subtype on the clinical, pathological, and prognostic features. They also determined that basal-like phenotype was linked to shorter overall and disease-free survival among 48 Japanese patients with TNBC over a median follow-up of 5.5 years.^27^ Clinicopathological features and outcomes of patients in basal and non-basal-like subgroups were compared in this study. The results of this comparison are shown in Table 3.

Yamamoto et al. demonstrated that basal-like tumors had a frequency of 45.8% in 48 patients with TNBC enrolled in the study.^27^ In a large cohort of women with triple-negative breast cancer (n=639) in British Columbia and Canada, 336 (52.6%) had basal-like subtype using the same criteria employed herein.^28^ Basal-like tumors among TNBC patients were significantly lower (Table 2), compared to other studies (51.7% versus 71%)^9^ but the results were very close.^27^^,^^28^

Among Iranian women with TNBC, EGFR+ tumors were associated with an increase in lymph node involvement (91.7% vs. 64.5%, p=0.045) that can be translated to worse prognosis in this subset of patients. In a study of 135 breast cancer patients, Sainsbury et al. demonstrated that EGFR, among different variables, is the single most important determinant of overall and relapse-free survival in lymph node-negative patients.^29^ Battaglia et al. also observed patients with metastatic breast tumors and axillary lymph node involvements are more likely to be EGFR+.^30^

## CONCLUSION

 The mean age of diagnosis of TNBC in Iran is not lower than the rest of the world; however, clinical stage, lymph node involvement and lymphovascular invasion were higher in Iranian patients. Like other studies, this survey demonstrated similar results for cancer recurrence during the follow-up period but the bone was the most common site of recurrence among Iranian patients.

In this study all clinicopathological findings, except one seen in EGFR positive basal-like tumors, were similar in our patients with basal-like and non-basal-like subtypes.

**Table 1:** Clinicopathological characteristics of patients with triple negative breast carcinoma

**Clinicopathological characteristics**	**Our study**	**Ref. 6**	**Ref. 3**	**Ref. 31**	**Ref. 32**
Age at diagnosis (years)	49.6 ± 11.7	52 ± 12	53	47.5	47.64
Tumor type, n (%) ductal medullary lobular	57 (95) 2 (3.3) 1 (1.7)	(93) - (2)	- - -	107 (92) 1 (<1) 1 (<1)	220 (86.3) - -
Mean tumor size (cm)	4.0 (3.0-6.0)	-	3	-	-
Nuclear grade, n (%) I II III	(0) 26 (43.3) 34 (56.7)	I, II (14) - (83)	(9.8) (24.2) (66.0)	(0) 17 (15) 98 (84)	2 (0.8) 29 (11.4) 217 (85.1)	
Lymph node involvement	42 (70.0)	(38)	87 (54.4)	62 (54)	160 (62.7)
Number of nodes involved	2.0 (0.0-6.0)	-	-	-	-
Neural invasion, n (%)	32 (53.3)	-	-	-	-
Lymphovascular invasion, n (%)	38 (63.3)	(26)	65 (39.6)	-	-
Recurrence and metastases, n (%) Bone Lung Brain Liver Local Recurrence Metastatic at presentation	11 (39.3) 5 (17.9) 3 (10.7) 3 (10.7) 4 (14.3) 2 (7.1)	- - - - - -	- - - - - -	28 (24) 48 (41) 16 (14) 34 (29) 45 (39) 13 (11)	10 (13) All visceral metastases 59 (74) - -
Disease stage, n (%) I II III IV	2 (3.3) 28 (46.7) 28 (46.7) 2 (3.3)	(33) (50) (18) -	- - - -	21 (18) 49 (42) 33 (28) 13 (11)	- - - -
follow-up duration month Death during follow-up, n (%) Disease-free interval Median month	24.0 7 (11.76) 21.13	- - -	- - -	34.1 - 19.9	- - -

**Table 2:** Immunohistochemistry results of triple negative breast carcinomas

**Type**	**No of patients (%)**
Basal-like	31 (51.7)
EGFR+ CK5/6-	4 (12.9)
EGFR- CK5/6+	19 (61.3)
EGFR+ CK5/6+	8 (25.8)
Non basal-like	29/60 (48.3)

**EGFR:** Epidermal growth factor receptor, **CK 5/6:** Cytokeratin 5/6

**Table 3:** Comparison between basal-like and non basal-like triple negative breast carcinomas

	**Basal-like**	**Non basal-like**	**p-value**
Age at diagnosis (years)	50.1 ± 13.0	49.0 ± 10.4	0.729
Tumor type, n (%) Intra-ductal carcinoma Medullary carcinoma Invasive Lobular Carcinoma	30 (96.8) 1 (2.2) 0 (0.0)	27 (93.2) 1 (3.4) 1 (3.4)	0.579
Tumor size (centimeter)	4.0 (3.5-5.0)	4.0 (2.5-6.0)	0.727
Tumor grade II III	12 (38.7) 19 (61.3)	14 (48.3) 15 (51.7)	0.603
Lymph node involvement, n (%)	24 (77.4)	18 (62.1)	0.262
Number of nodes involved	3.0 (1.0-6.0)	2.0 (1.0-6.5)	0.635
Neural invasion, n (%)	18 (58.1)	14 (48.3)	0.605
Vascular invasion, n (%)	21 (67.7)	17 (58.6)	0.593
Distant metastases, n (%)	8 (25.8)	8 (27.6)	1.000
Disease stage I II III IV	0 (0.0) 15 (48.4) 16 (51.6) 0 (0.0)	2 (6.9) 13 (44.8) 12 (41.4) 2 (6.9)	0.199

The exception was associated with an increased incidence of lymph node involvement and a worse prognosis in this subset of patients.
